# National record linkage study of mortality for a large cohort of opioid users ascertained by drug treatment or criminal justice sources in England, 2005–2009

**DOI:** 10.1016/j.drugalcdep.2014.09.782

**Published:** 2015-01-01

**Authors:** Matthias Pierce, Sheila M. Bird, Matthew Hickman, Tim Millar

**Affiliations:** a Institute of Brain Behaviour & Mental Health, Faculty of Medical and Human Sciences, University of Manchester; b Medical Research Council, Cambridge; University of Strathclyde; c School of Social and Community Medicine, University of Bristol

**Keywords:** Mortality, Opioid use, Addiction epidemiology, Drug related poisoning mortality, Ageing opioid users

## Abstract

**Background:**

Globally, opioid drug use is an important cause of premature mortality. In many countries, opioid using populations are ageing. The current study investigates mortality in a large cohort of opioid users; with a focus on testing whether excess mortality changes with age.

**Methods:**

198,247 opioid users in England were identified from drug treatment and criminal justice sources (April, 2005 to March, 2009) and linked to mortality records. Mortality rates and standardised mortality ratios (SMRs) were calculated by age-group and gender.

**Results:**

There were 3974 deaths from all causes (SMR 5.7, 95% Confidence Interval: 5.5 to 5.9). Drug-related poisonings (1715) accounted for 43% of deaths. Relative to gender-and-age-appropriate expectation, mortality was elevated for a range of major causes including: infectious, respiratory, circulatory, liver disease, suicide, and homicide. Drug-related poisoning mortality risk continued to increase beyond 45 years and there were age-related increases in SMRs for specific causes of death (infectious, cancer, liver cirrhosis, and homicide). A gender by age-group interaction revealed that whilst men have a greater drug-related poisoning mortality risk than women at younger ages, the difference narrows with increasing age.

**Conclusion:**

Opioid users’ excess mortality persists into old age and for some causes is exacerbated. This study highlights the importance of managing the complex health needs of older opioid users.

## Highlights

• Opioid user mortality is almost 6 times higher than in the general population.
• Mortality is elevated for a range of diseases and for homicide and suicide.
• Excess mortality persists into old age and for some causes is exacerbated.
• Drug-related poisoning (DRP) accounts for just under a half of opioid user deaths.
• Young female DRP mortality is lower than male but the difference narrows with age.

## 1 Introduction

Illicit drug use, especially opioid addiction, is an acknowledged public health problem in most developed countries and a growing problem worldwide (Degenhardt and Hall, 2012; Degenhardt et al., 2004; Lim et al., 2013). Deaths due to illicit drug use are an important, increasing (Murray et al., 2013), and preventable cause of premature mortality (Bargagli et al., 2006). Recent global estimates suggest that the years of life lost due to illicit drugs are greater than for alcohol, because the former tend to occur at an earlier age (Degenhardt and Hall, 2012). In England and Wales, deaths directly attributed to illicit drug use (i.e., drug related poisonings) account for 12% of all fatalities between 16 and 40 years of age (Office for National Statistics Statistical Bulletin, 2013). The risk of a drug related poisoning is higher for males, drug injectors and those with concurrent depressant use (Davoli et al., 2007; Degenhardt et al., 2011; Merrall et al., 2012). A substantial, international body of evidence demonstrates excess mortality risk for many causes of death including: suicide; homicide; infectious disease; and liver-related disease (Bird, 2010; Crump et al., 2013; Degenhardt et al., 2014; Ghodse et al., 1998; Merrall et al., 2012).

Statistically powerful studies are required to ascertain which specific causes of death are elevated and to identify the key behavioural and demographic risk factors. Large scale, preferably national, record linkage studies can address the problem of statistical power, but often exclude non-treatment-seeking individuals. A recent overview by Degenhardt et al. (2011) identified the exclusion of non-treatment-seeking users and the paucity of national studies as important limitations of the current literature, that constrain the capacity to generalise findings to the wider population of opioid users.

The ageing population of opioid users is an emerging issue for treatment services in many developed countries (Gossop, 2008; Gfroerer et al., 2003). Whilst some studies describe poor health among older opioid users (Hser et al., 2004; Rosen et al., 2008), few have investigated mortality according to age-group (Degenhardt et al., 2011). Degenhardt et al. (2014) recently reported changes in the distribution of causes of death with age among 43,789 treated opioid users observed over a 20 year period. They highlight that disease, such as cancer or liver disease, accounts for an increasing proportion of deaths with increasing age, as seen in the general population, but do not provide a measure of how opioid users’ excess mortality changes with age (i.e., by reference to what would be expected in the general population).

The study reported here uses data for a large, national opioid user cohort that includes both treatment and non-treatment seeking individuals. In this study we aim to: (i) describe excess mortality due to all-causes and drug-related poisonings; (ii) identify specific causes of death which are elevated; (iii) assess whether cause-specific mortality risk compared to the general population persists, decreases or increases with age; (iv) assess whether the difference in drug related poisoning mortality risk between male and female opioid users persists with age.

The cohort was drawn from the Drug Data Warehouse (Millar et al., 2012), an anonymous, case-linked collection of secondary datasets about substance (drug use and/or alcohol misuse) users in England and Wales. The Drug Data Warehouse includes data from: drug treatment services; prison and probation services; criminal justice referral; and drug testing on arrest schemes. Internationally, this is the largest opioid user cohort (*n* = 198,247) for whom mortality by specific cause has been reported. The inclusion of treatment and non-treatment-seeking individuals in a national cohort is both necessary and novel. This, combined with a focus on age effects and the necessary statistical power to investigate these, addresses key limitations identified (Degenhardt et al., 2011) in the literature to date.

## 2 Methods

### 2.1 Population, cohort, and data linkage

Data were extracted from the Drug Data Warehouse for a cohort of opioid users, aged 18 to 64 years, actively using or being treated for opioid use, in England over the period 1st April 2005 to 31st March 2009. Deaths occurring in the cohort were established by case linkage to national mortality records.

Table 1 shows case definitions for cohort inclusion from each data source. All data sources provided details of age group and gender.

Record linkage between criminal justice and treatment data sources used an identifier based on initials, date of birth, and gender. Additional data available from criminal justice sources provided evidence of duplicated identifiers for 23% of cases (*n* = 58,547 of 256,794); conservatively, all records for these were excluded from the current analysis. Case-linkage to mortality records used the additional criterion of region of residence, to increase accuracy. Linked mortality records (*n* = 478) were removed where evidence of new treatment or criminal justice activity, after the apparent date of death, indicated an erroneous match.

Data were rendered anonymous to the research team, via irreversible encryption of identifying information, prior to their release by source organisations.

### 2.2 Mortality records

The Office for National Statistics provided records of deaths of persons aged 18–64 years occurring during the observation period 1st April, 2005 to 31st March, 2009 and registered by 30th September, 2011. This allowed for late registration of deaths referred for investigation by a coroner (Bird, 2013). Underlying cause was recorded for each death and coded according to the World Health Organisation's International Classification of Disease 10th revision (ICD-10; WHO, 2011).

### 2.3 Population data and classification of cause of death

Population mortality rates were compiled from mid-year population estimates and published figures on deaths registered between 2005 and 2008 for England and Wales (Office for National Statistics Statistical Bulletin, 2011) as published data were not available by death-year for the exact observation period. Rates were calculated by gender, five year age-group (15–19, 20–24, 25–29, 30–34, 35–39, 40–44, 45–49, 50–54, 55–59, 60–64), and for underlying cause of death.

Drug-related poisoning deaths were defined according to the Office of National Statistics UK harmonised definition (Office for National Statistics Statistical Bulletin, 2013) which uses the following ICD-10 codes: F11-16, F18-19 (mental and behavioural disorders due to psychoactive substance use, excluding alcohol and tobacco); X40-44 (external causes: accidental poisoning by drugs, medicaments and biological substances); X60-64 (external causes: intentional self-poisoning drugs, medicaments and biological substances); X85 (external causes: assault by drugs, medicaments and biological substances); Y10-14 (external causes: poisoning by drugs, medicaments and biological substances undetermined intent).

Suicides include deaths of ‘undetermined intent’ (ICD-10: X60-X84; Y10-Y34). To avoid double counting, drug-related poisoning mortality was treated as a separate category and were excluded from reporting of the ICD-10 Chapters ‘external causes’ and ‘mental and behavioural disorders’.

### 2.4 Approvals

Use of official mortality records was approved by the Office for National Statistics Microdata Release Panel. Use of data from the Drug Data Warehouse was authorised by those organisations providing data. The NHS Central Office for Research Ethics Committees and The University of Manchester Research Ethics Committee advised that further approval was not required for a study of this type.

### 2.5 Statistical analysis

*Description of measures used: Crude mortality rate (CMR):* Describes the rate of death occurring in the cohort. A CMR of 73 per 10,000 pys translates to 73 deaths occurring among 10,000 people over a one-year period or to 73 deaths occurring among 20,000 people over a 6-month period. *Standardised Mortality Ratio (SMR)*: describes the extent to which mortality in a cohort differs from that which would be seen in an ‘average’ population, matched for age and gender. An SMR of 5.7 means that there were 5.7 times more deaths occurring in the cohort than would have occurred in a sample of the general population who had the same distribution of age and gender.

Crude mortality rates (CMR) per 10,000 person-years (pys) were calculated for all-cause mortality, and drug-related poisoning deaths. An individual's risk period began at the date of their earliest observation in the cohort on or after 1st April 2005 and ceased at the end of data collection (31st March, 2009) or the date of death, if earlier. Individuals already in treatment on 1st April, 2005 began their time at risk from that date.

Observed deaths (O) were compared to gender and age appropriate expected mortality (E) to derive standardised mortality ratios (SMR = O/E). The expected mortality was calculated by multiplying the (disease specific) mortality rate observed in the general population by the person years of follow-up seen in the analysis cohort, matched by age and gender (indirect method). Confidence intervals for CMRs and SMRs were calculated using a normal approximation to the Poisson distribution for the observed number of deaths. All *p* values are two sided.

Following strong prior information for drug-related poisonings (King et al., 2012, 2013), we assessed whether mortality differences between males and females persist with age by testing for an interaction between gender and age-group. The interaction was evaluated by testing whether the relative risk, comparing the drug-related poisoning mortality rate between males and females, was equal across age groups, using a chi-squared test. Evidence for presence of an interaction was set at *p* < 0.01. As a sensitivity analysis, we assessed whether evidence for an age and gender interaction was due to differences in behavioural risk factors by carrying out an adjusted analysis on the subcohort of treated individuals for whom we have information available on risk factors (*n* = 151,983). This is described in the supplementary material^1^.

Cause-specific CMRs and SMRs were first calculated at the ICD-10 Chapter level. To retain statistical power the a priori analysis strategy was to present CMR/SMRs at subsequent ICD-10 lower, more detailed descriptive levels if (a) the SMR for the higher level was ≥5; and (b) the expected number of deaths for the lower level was ≥5. Four additional causes of death were considered at the lower descriptive level due to their importance in the literature: suicide, viral hepatitis, liver cancer, and human immunodeficiency virus (HIV) (Darke et al., 2007; Darke and Ross, 2002; Degenhardt et al., 2011; Merrall et al., 2012).

The relationship between cause-specific SMR and age was investigated, for the entire cohort, using Poisson regression models. Analyses were undertaken using Stata version 13.

## 3 Results

### 3.1 All-cause and drug-related poisoning mortality (Table 2)

The cohort (*n* = 198,247) contributed 541,891 pys of follow up: the median follow-up time was 3.1 years (Inter Quartile Range (IQR): 1.7 to 4 years). The median age at cohort entry was 32.1 years (IQR: 26.4 to 38.7 years), 142,608 (72%) cohort members were male and 184,256 (93%) were identified as heroin users (as opposed to users of other opioids).

There were 3974 deaths from all causes with a CMR of 73 deaths (95% confidence interval 71 to 76) per 10,000 pys and an SMR of 5.7 (95% CI: 5.5 to 5.9); thus there were more than five and a half times the number of deaths than would be expected in the age and gender appropriate general population.

Drug-related poisonings (CMR 32; 95% CI 30 to 33) were the most common cause of mortality, accounting for 43% of deaths.

### 3.2 All-cause and drug-related poisoning mortality by age and gender (Table 3)

Male all-cause CMR was higher than for females (81 vs. 54, *p* < 0.001) but males’ SMR was lower (5.5 vs. 6.9, *p* < 0.001), reflecting lower female mortality in the general population.

Tables 2–3.

Male drug-related poisoning CMR (35; 95% CI 34 to 37) was substantially higher than for females (23; 95% CI 21 to 25, *p* < 0.001).

Across gender, drug-related poisoning CMR increased markedly with age, from 19 (95% CI 16 to 23) at 18–34 years to 45 (95% CI 40 to 50) at 45–64 years (*p* < 0.001) and was higher at 45–64 than 35–44 years (*p* = 0.04).

There was clear evidence to reject the hypothesis that the male vs. female comparison in drug-related poisoning rate was equivalent for different age-groups (chi squared (2 dof) = 13.04, *p* = 0.002). This interaction revealed that males had almost double the drug-relating poising CMR compared to females at 18–34 years (29; 95% CI 26 to 31 vs. 15; 95% CI 13 to 18) but this difference narrows considerably with age (i.e. at 45–64: 47; 95% CI 41 to 53 vs. 40; 95% CI 32 to 51). This interaction also revealed that there was a clear difference in drug related poisoning rates at age 35–44 (relative risk males vs. females 1.3, 95% CI 1.1 to 1.5) but this was less apparent at age 45–64 (relative risk 1.2, 95% CI 0.9 to 1.5).

### 3.3 Cause-specific mortality (Table 4a)

CMRs were higher than expected for all ICD-10 classifications, except ‘other’ causes. Chapter level SMRs ranged from 1.7 (95% CI 1.3 to 2.3, nervous system diseases) and 1.8 (95% CI 1.6 to 2.0, cancers) to 12.6 (95% CI 10.8 to 14.8, infectious/parasitic disease) and 17.2 (95% CI 11.0 to 27.0, skin/subcutaneous tissue disease). The latter included five deaths from abscesses and seven from cellulitis.

After drug-related poisoning deaths, ‘external causes’ (with drug-related poisonings excluded) were the most frequent cause of mortality (21% of all deaths; CMR 8.9; 95% CI 8.1 to 9.7), notably suicide (drug-related poisonings excluded, 5%) and homicide (2%). Rates of homicide were 12 times higher (SMR 12.2; 95% CI 9.8 to 15.3) and suicide (where not also classified as drug-related poisonings) three times higher (SMR 2.9; 95% CI 2.5 to 3.4) than expected. With drug-related poisonings included, the SMR for suicides was 4.3 (95% CI 3.9 to 4.8).

Circulatory and digestive system diseases accounted for similar proportions of deaths (both 11%) but with markedly different SMRs (3.1; 95% CI 2.8 to 3.4 vs. 6.4; 95% CI 5.9 to 7.1). Digestive system mortality was due mainly to diseases of the liver. Respiratory system disease was also common, accounting for 7% (CMR 4.8; 95% CI 4.2 to 5.4) with an SMR of 8.9 (95% CI 7.9 to 10.1): half of respiratory system deaths were due to chronic lower respiratory disease and a further 39% to influenza and pneumonia.

Fifteen per cent of the 2259 deaths not categorised as drug-related poisonings were caused by liver disease (*n* = 345); the majority alcoholic liver disease (72%) or fibrosis and cirrhosis of the liver (19%), the latter associated with SMR of 9.6 (95% CI 7.5 to 12.2). Additionally, liver cancer accounted for 38 deaths (SMR 9.2; 95% CI 6.7 to 12.7).

### 3.4 Cause-specific mortality by age (Table 4b)

For circulatory, respiratory and digestive system disease, CMRs, as to be expected, increased sharply with age. At 35–44 years, CMRs were highest for circulatory (8.1; 95% CI 6.9 to 9.6) and digestive system disease (9.3; 95% CI 8.0 to 10.9) but, by 45–64 years, cancer (28.0; 95% CI 24.3 to 32.2), circulatory (29.9; 95% CI 26.0 to 34.3) and digestive system (29.3; 95% CI 25.5 to 33.6) deaths dominated, with respiratory deaths close behind (19.2; 95% CI 16.2 to 22.8).

Table 4a.

Table 4b.

The SMR increased markedly with age for infectious/parasitic disease (5.7; 95% CI 3.8 to 8.6 at 18–34 years to 23.2; 95% CI 18.6 to 28.8 at age 45–64 years, trend *p* = <0.001), cancers (1.3; 95% CI 0.9 to 1.8 vs. 2.1; 95% CI 1.8 to 2.4, trend *p* = 0.003), and liver fibrosis and cirrhosis (2.6; 95% CI 0.7 to 10.5 vs. 14.1; 95% CI 10.6 to 18.9, trend *p* < 0.001), but not for other specific disease causes.

For homicide, CMR changed little with age but the SMR increased markedly (*p* = 0.002) from 8.8 (95% CI 6.3 to 12.2) at 18–34 years to 27 (95% CI 16 to 46) at 45–64 years; thus, older opioid users were very much more likely to be the victims of homicide than their counterparts in the general population. Risk of suicide (drug-related poisoning excluded) was elevated for all age groups, but SMRs showed no trend with age (*p* = 0.55).

## 4 Discussion

### 4.1 Principal findings

Consistent with previous research, all-cause mortality for England's opioid user cohort was highly elevated (SMR 5.7; 95% CI 5.5 to 5.9). Although drug-related poisoning was the predominant cause, the cohort had elevated risks for all main causes of death.

Our findings are highly informative for the evidence-base on age-related mortality in active opioid users because 15% of pys, 18% of drug-related poisoning deaths and 32% of all fatalities occurred at 45–64 years. Importantly, this study is the first to calculate age trends in excess deaths and we demonstrate that health inequalities persist with increasing age and for some disease causes widen (notably, infectious/parasitic, cancer, fibrosis and cirrhosis of the liver). This is likely, in part, due to synergistic effects, including the well-known combination of viral hepatitis and alcohol (Fu et al., 2007; Hutchinson et al., 2005; Marrero et al., 2005; McDonald et al., 2011). The SMR, but not CMR, for homicide also increased markedly with age, indicating an age persistent risk not present in the general population.

Our analysis provides the first demonstration of a highly significant, age-related increase in opioid users’ drug-related poisoning mortality rate that persists beyond 45 years of age. This finding contrasts with routine reports of drug-related poisoning death rates per million of population in England and Wales (Office for National Statistics Statistical Bulletin, 2013) which indicate a peak rate at 30–39 years. However, official reporting does not take into account differential user prevalence by age-group.

External causes, excluding drug-related poisonings, accounted for three times the number of deaths expected (SMR 3.3; 95% CI 3.0 to 3.6). Homicide and suicide were major causes, the latter resonating with a recent policy focus on the contribution of drug dependency to suicide (Department of Health, 2012). The SMR for suicide remained elevated when the subset of drug-related suicides (which may include misclassified accidental overdoses) was excluded.

The drug-related poisoning mortality risk among female opioid users was lower than for men, but the gender difference was considerably more marked among those aged under 35 years. As a sensitivity analysis we established that, for the treatment-seeking sub-cohort, this interaction was still evident once we adjusted for reported behavioural risks, of injecting, problematic use of alcohol, or benzodiazepines (test for interaction *p* < 0.001; see supplementary material). Our data do not support speculation on the potential underlying causal effects at work here, but underscore a need for relevant explanatory studies. A potential avenue is the observation (Kimber et al., 2010) that females may cease drug use at an earlier stage: use persisting into older age may be more severely problematic, but the effect may be manifested to a greater extent among women.

### 4.2 Comparison with other key studies

The excess risk reported here was considerably less than that reported in a meta-analysis of the international literature (SMR 14.7; 95% CI 12.8 to 16.5) (Degenhardt et al., 2011). This may reflect lower HIV prevalence and better treatment access in England compared with many countries; or earlier epochs. The SMR for the England cohort was slightly lower than that observed for an earlier (1985 to 2005) Australian treatment cohort (SMR 6.5; 95% CI 6.3 to 6.7) (Degenhardt et al., 2014). It was slightly higher than for the most readily comparable, near contemporary (2001–2006), geographically proximal, Scottish drug treatment cohort (SMR 4.8; 95% CI 4.6 to 5.0) (Merrall et al., 2012). This may reflect the latter's inclusion of non-opioid users (35%), despite a higher proportion injecting (48%), and younger age, demonstrating the importance of considering the full range of salient factors when comparing cohorts’ SMRs.

### 4.3 Strengths and limitations

Steps were taken to minimise false positive data linkages by comparing minimal identifiers with unique criminal justice system (CJS) identifiers, removing all cases for which there was evidence of a potentially non-unique minimal identifier. This approach applied to the 73% of identifiers that had a unique CJS identifier and was conservative, insofar as these CJS identifiers may themselves be subject to transcription errors as a consequence of manual data entry. However, some misclassification and failures-to-match may remain. The use of self-report may underestimate levels of behavioural risks (see Supplementary material^2^). There was an absence of active follow up and so any cessation of declared behavioural risks was not accounted for; the use of a short median follow up time, however, limits any resultant bias. Additional factors contributing to excess mortality, and common amongst this group, were not measured, including: high rates of smoking, high levels of alcohol consumption that is not acknowledged as problematic, low socioeconomic status, low quality of life, high rates of depression and co-morbidity, and poor diet (Copeland et al., 2012). It is also important to note that whilst our findings should inform management of older, active, opioid users, we are unable to make inferences about longer-term mortality outcomes for those who desist from use at a younger age, although this may not be the norm (Termorshuizen et al., 2005; Hser et al., 2004). Treatment effects on mortality risk were not considered here but are being investigated in parallel work. Finally, although the cohort was derived from multiple national data sources it does not, of course, represent all opioid users. Those users not identified in either treatment or the criminal justice systems may be less problematic and at lower risk. Whilst it is difficult to study this hidden population, future work could, potentially, explore the extent to which cases of fatal opioid-related poisoning have prior criminal justice or treatment contact, as is done routinely in Scotland (Hecht et al., 2014).

Despite these limitations, the inclusion of users accrued from national, treatment and non-treatment sources, with a focus on age effects, serves to address important limitations in the existing literature identified by others (Degenhardt et al., 2011). The statistical power provided by such a large cohort, more than double the largest to date (Crump et al., 2013; Degenhardt et al., 2014; Ghodse et al., 1998; Merrall et al., 2012), strengthens previous research internationally, particularly in respect of deaths not directly attributed to opioid misuse.

### 4.4 Conclusions and implications

The opioid misusing population in the UK, and elsewhere, is ageing (Royal College of Psychiatrists, 2011); many of those who initiated use during the heroin ‘epidemics’ of the 1980s and 1990s, and who have not died or abstained, are now in their fourth or fifth decade of life. We demonstrate that health inequalities between opioid users and the general population persist and, for some diseases, widen with age. These findings underline the importance for public health policy and treatment providers of delivering effective addiction treatment for older age groups, who are characterised by multiple and complex health problems. Importantly, as the opioid using population ages, so their risk of death due to drug-related poisoning is likely to increase: national targets need to adjust for age in order effectively to monitor the impact of policies with the aim of reducing drug-related poisoning deaths. Crucially, the new health information on drug-related poisoning mortality risk in older age as presented here should be promoted to opioid users themselves, to emphasise that their risk of overdose does not decline, but rather increases, with age. The increased SMRs with age for homicide and cancer (in addition to infectious diseases and liver fibrosis/cirrhosis) also merit attention.

## Author disclosures

### Role of the funding source

The research was funded by a grant from the Medical Research Council (MRC grant number G1000021), provided within the RCUK Addiction Research Strategy. The MRC had no further role in study design; in the collection, analysis and interpretation of data; in the writing of the report; or in the decision to submit the paper for publication. Public Health England, The Home Office, and The Office for National Statistics have been provided with a pre-submission version of this manuscript but have not exerted any editorial control over, or commented on, its content.

### Contributors

Millar and Bird conceived of the study. Pierce with input from Bird wrote the analysis plan. Pierce analysed the data and wrote a first draft of the manuscript. Millar, Bird and Hickman supervised data analysis. All interpreted the data and edited the manuscript.

### Conflict of interest

Millar has received research funding from the UK National Treatment Agency for Substance Misuse and the Home Office. He is a member of the organising committee for, and chairs, conferences supported by unrestricted educational grants from Reckitt Benckiser, Lundbeck, Martindale Pharma, and Britannia Phamaceuticals Ltd, for which he receives no personal remuneration. Bird holds GSK shares, is an MRC programme leader. She chaired Home Office's Surveys, Design and Statistics Subcommittee (SDSSC) when SDSSC published its report on 21st Century Drugs and Statistical Science. She has previously served as UK representative on the Scientific Committee for European Monitoring Centre for Drugs and Drug Addiction. She is co-principal investigator for MRC-funded, prison-based N-ALIVE pilot Trial.

## Figures and Tables

**Table 1:** Data sources and selection criteria used to define the opioid user cohort.

Data source	Cohort population	Case definition for opioid use	Number of individuals
National drug treatment monitoring system	Treatment clients receiving community or residential structured addiction treatment	Reporting opioid use at triage to treatment	47,707
Drug test on arrest	Subjects arrested following a “trigger” offence, or at police discretion, and then tested for opioids and cocaine metabolites by saliva sample, in areas where the drug test on arrest programme operates	Testing positive for opiate use (indicative of recent use)	54,937
Drug intervention record	Persons assessed for referral to structured drug treatment following identification in a criminal justice setting	Reporting weekly or greater opioid use at assessment	39,517
Offender assessment system	Offenders assessed in prison, or whilst on probation for needs and risks prior to sentencing, and at various points throughout their sentence, including assessment of substance use	Reporting weekly or greater opioid use at assessment	151,983

**Table 2:** Description of opioid user cohort: age, gender, and mortality.

Number of individuals	198,247
Person-years under observation	541,891
Median age at cohort accession date, years [IQR]	32.1 [26.4, 38.7]
Gender (%)	
Male	142,608 (72)
Female	55,639 (28)
Age group at cohort entry (%)	
18–24	37,172 (19)
25–34	85,725 (43)
35–44	55,005 (28)
45–64	20,345 (10)
All-cause mortality	
Number of deaths	3,974
Rate per 10,000 pys [95% CI]	73 [71,76]
Expected deaths	695
SMR [95% CI]	5.7 [5.5, 5.9]
Drug-related poisoning death	
Number of deaths	1715
Rate per 10,000 pys [95% CI]	32 [30, 33]

**Table 3:** All-cause crude mortality rates, standardised mortality ratios and drug-related poisoning mortality rates by age and gender: opioid user cohort of 198,247 individuals and 541,891 person years.

Variable	All-cause mortality						Drug-related poisoning deaths	
	Pys, thousand	Deaths (*n*)	CMR, per 10,000 pys [95% CI]		Expected deaths (*n*)	SMR [95% CI]	Deaths (*n*)	CMR, per 10,000 pys [95% CI]
Gender								
Male	383	3,118	81	[79,84]	572	5.5 [5.3, 5.6]	1,353	35 [34,37]
Female	159	856	54	[50,57]	123	6.9 [6.5, 7.4]	375	23 [21,25]

Age-group, time updated								
18–24	63	185	30	[26,34]	32	5.7 [5.0, 6.6]	119	19 [16,23]
25–34	236	1,052	45	[42,47]	176	6.0 [5.6, 6.4]	602	26 [24,28]
35–44	175	1,480	84	[80,89]	233	6.3 [6.0, 6.7]	686	39 [36,42]
45–64	68	1,257	184	[174,194]	254	5.0 [4.7, 5.2]	308	45 [40,50]

Age-group males only, time updated								
18–34	203	954	47	[44,50]	174	5.5 [5.2, 5.9]	580	29 [26,31]*
35–44	128	1,148	89	[84,95]	192	6.0 [5.7, 6.4]	534	42 [38,45]*
45–64	51	1,016	199	[187,212]	207	4.9 [4.6, 5.2]	239	47 [41,53]*

Age-group females only, time updated								
18–34	95	283	30	[27,34]	34	8.3 [7.3, 9.3]	141	15 [13,18]*
35–44	47	332	71	[63,79]	42	7.9 [7.1, 8.8]	152	32 [28,38]*
45–64	17	241	140	[123,158]	47	5.1 [4.5, 5.8]	69	40 [32,51]*

* Males vs. females relative risk at levels of age-group: 18–34: 1.9 (95% CI: 1.6 to 2.3); 35–44: 1.3 (95% CI: 1.1 to 1.5); 45–64: 1.2 (95% CI: 0.9 to 1.5). Test for equality of relative risks: chi squared (2 dof) = 13.04; *p* = 0.002.

**Table 4a:** Cause-specific CMRs and SMRs for the opioid user cohort of 198,247 individuals and 541,891 person years.

ICD 10 level	Disease description	ICD-10 codes*	Observed deaths	CMR, per 10,000 pys [95% CI]		Expected deaths	SMR [95% CI]	
Chapter	Infectious/parasitic	A00-B99	159	2.9	[2.5, 3.4]	12.6	**12.6**	[10.8, 14.8]
3	Viral hepatitis	B15-B19	82	1.5	[1.2, 1.9]	1.4	**57.2**	[46.1, 71.1]
3	HIV	B20-B24	31	0.6	[0.4, 0.8]	4.4	**7.0**	[5.0, 10.0]

Chapter	Cancers	C00-D48	296	5.5	[4.9, 6.1]	166.3	**1.8**	[1.6, 2.0]
	Liver cancer	C22	38	0.7	[0.5, 0.9]	4.1	**9.2**	[6.7, 12.7]
Chapter	Endocrine	E00-E90	29	0.5	[0.4, 0.8]	12.5	**2.3**	[1.6, 3.3]
Chapter	Mental and behavioural*	F00-F99	31	0.6	[0.4, 0.8]	7.2	**4.3**	[3.0, 6.1]
Chapter	Nervous system	G00-G99	47	0.9	[0.7, 1.2]	27.4	**1.7**	[1.3, 2.3]
Chapter	Circulatory system	I00-I99	418	7.7	[7.0, 8.5]	134.1	**3.1**	[2.8, 3.4]
Chapter	Respiratory system	J00-J99	259	4.8	[4.2, 5.4]	29.0	**8.9**	[7.9, 10.1]
3	Influenza and pneumonia	J09-J18	102	1.9	[1.6, 2.3]	11.6	**8.8**	[7.2, 10.7]
3	Chronic lower respiratory diseases	J40-J47	130	2.4	[2.0, 2.8]	10.4	**12.6**	[10.6, 14.9]
Chapter	Digestive system	K00-K93	423	7.8	[7.1, 8.6]	65.7	**6.4**	[5.9, 7.1]
3	Diseases of liver	K70-K77	345	6.4	[5.7, 7.1]	49.5	**7.0**	[6.3, 7.8]
2	Alcoholic liver disease	K70	249	4.6	[4.1, 5.2]	37.1	**6.7**	[5.9, 7.6]
2	Fibrosis and cirrhosis	K74	66	1.2	[1.0, 1.6]	6.9	**9.6**	[7.5, 12.2]
Chapter	Skin and subcutaneous tissue	L00-L99	19	0.4	[0.2, 0.5]	1.1	**17.2**	[11.0, 27.0]
Chapter	Musculoskeletal system and connective tissue	M00-M99	12	0.2	[0.1, 0.4]	2.7	**4.5**	[2.6, 7.9]
Chapter	External causes**	V01–Y98	482	8.9	[8.1, 9.7]	146.3	**3.3**	[3.0, 3.6]
3	Homicide***	X86-Y09	77	1.4	[1.1, 1.8]	6.3	**12.2**	[9.8, 15.3]
3	*Suicide excluding drug-related poisonings*	*X65-X84 & Y15-Y34*	*199*	*3.7*	*[3.2, 4.2]*	*68.2*	***2.9***	*[2.5, 3.4]*
	*Suicide including drug-related poisonings*	*X60-X84 & Y10-Y34*	*351*	*6.5*	*[5.8, 7.2]*	*81.9*	***4.3***	*[3.9, 4.8]*
Chapter	Not classified elsewhere****	–	66	1.2	[1.0, 1.6]	12.2	**5.4**	[4.3, 6.9]
–	Other*****		18	0.3	[0.2, 0.5]	19.6	**1.0**	[0.6, 1.5]

* Excluding 916 categorised as drug-related poisonings (ICD10 codes: F11-16; F18-19).

** Excluding 799 categorised as drug-related poisonings (6 homicides and 152 suicides: ICD10 codes X40-X44, X60-X64, X85, Y10-Y14.

*** Including 14 cases of ‘accelerated registration’ where the coroner's inquest is adjourned until legal proceedings are completed.

**** Including 61 cases of ‘other ill-defined and unspecified causes of mortality’.

***** Other deaths: Congenital malformations, deformations and chromosomal abnormalities (*n* = 8); diseases of: the blood (*n* = 6); the genitourinary system (*n* < 5); pregnancy, childbirth and the pueriperium (*n* < 5) and conditions originating in the perinatal period (*n* < 5).

**Table 4b:** Cause-specific CMRs and SMRs by age at risk for the opioid user cohort of 198,247 individuals and 541,891 person years.

	Age-group								
Cause of death and SMR linear trend by age-group	18–34 years, (pys = 298,111)			Age-group:35–44 years, (pys = 175,464)			45–64 years, (pys = 68,317)		
	*n*†	CMR per 10k pys [95% CI]	SMR [95% CI]	*n*†	CMR per 10k pys [95% CI]	SMR [95% CI]	*n*†	CMR per 10k pys [95% CI]	SMR [95% CI]
Infectious/parasitic diseases SMR trend *p* < 0.001	23	0.8 [0.5, 1.2]	**5.7** [3.8, 8.6]	56	3.2 [2.5, 4.1]	**11.0** [8.5, 14.3]	80	11.7 [9.4, 14.6]	**23.2** [18.6, 28.8]
Cancers SMR trend *p* = 0.003	33	1.1 [0.8, 1.6]	**1.3** [0.9, 1.8]	72	4.1 [3.3, 5.2]	**1.5** [1.2, 1.9]	191	28.0 [24.3, 32.2]	**2.1** [1.8, 2.4]
Circulatory system SMR trend *p* = 0.24	71	2.4 [1.9, 3.0]	**3.6** [2.9, 4.6]	143	8.1 [6.9, 9.6]	**3.2** [2.7, 3.8]	204	29.9 [26.0, 34.3]	**2.9** [2.5, 3.3]
Respiratory system SMR trend *p* = 0.24	40	1.3 [1.0, 1.8]	**7.5** [5.5, 10.2]	88	5.0 [4.1, 6.2]	**10.2** [8.3, 12.6]	131	19.2 [16.2, 22.8]	**8.7** [7.3, 10.3]
Digestive system SMR trend *p* = 0.16	59	2.0 [1.5, 2.6]	**5.8** [4.5, 7.5]	164	9.3 [8.0, 10.9]	**6.0** [5.1, 6.9]	200	29.3 [25.5, 33.6]	**7.1** [6.2, 8.2]
Diseases of liver: SMR trend *p* = 0.10	44	1.5 [1.1, 2.0]	**6.4** [4.7, 8.5]	136	7.8 [6.6, 9.2]	**6.3** [5.3, 7.4]	165	24.2 [20.7, 28.1]	**7.9** [6.8, 9.2]
Alcoholic liver disease SMR trend *p* = 0.75	35	1.1 [0.8, 1.6]	**6.8** [4.9, 9.4]	105	6.0 [4.9, 7.2]	**6.4** [5.3, 7.7]	109	16.0 [13.2, 19.3]	**7.1** [5.9, 8.5]
Fibrosis and cirrhosis of liver SMR trend *p* < 0.001	2	0.1 [0.0, 0.3]	**2.6** [0.7, 10.5]	18	1.0 [0.6, 1.6]	**6.3** [3.9, 9.9]	46	6.7 [5.0, 9.0]	**14.1** [10.6, 18.9]
Homicides SMR trend *p* = 0.002	35	1.2 [0.8, 1.6]	**8.8** [6.3, 12.2]	28	1.6 [1.1, 2.3]	**15.3** [10.7, 22.3]	14	2.0 [1.2, 3.5]	**27.3** [16.1, 46.0]
Suicides SMR trend *p* = 0.55	93	3.1 [2.5, 3.8]	**2.7** [2.2, 3.3]	79	4.5 [3.6, 5.6]	**3.1** [2.5, 3.9]	27	4.0 [2.7, 5.8]	**3.2** [2.2, 4.6]

† Number of observed deaths.

## Footnotes

1 Supplementary material can be found by accessing the online version of this paper at http://dx.doi.org and by entering doi:..

2 Supplementary material can be found by accessing the online version of this paper at http://dx.doi.org and by entering doi:...
