# Corrigendum to “National record linkage study of mortality for a large cohort of opioid users ascertained by drug treatment or criminal justice sources in England, 2005–2009” [Drug Alcohol Depend. 146 (2015) 17–23]

**DOI:** 10.1016/j.drugalcdep.2015.09.016

**Published:** 2015-11-01

**Authors:** Matthias Pierce, Sheila M. Bird, Matthew Hickman, Tim Millar

**Affiliations:** a Institute of Brain Behaviour & Mental Health, Faculty of Medical and Human Sciences, University of Manchester, UK; b Medical Research Council, Cambridge, University of Strathclyde, UK; c School of Social and Community Medicine, University of Bristol, UK

The authors regret “In this paper, we reported on mortality for a cohort of opioid users ascertained by drug treatment or criminal justice sources in England. In Table 1, we reported the number of individuals accrued to the cohort from each setting. However, the original Table 1 contained an error, such that the alignment of settings and their associated counts were incorrect. The correct Table 1 appears below. This error does not alter the analysis or findings of the study.

The authors would like to apologise for any inconvenience caused.

**Table 1:** Data sources and selection criteria used to define the opioid user cohort.

Data source	Cohort population	Case definition for opioid use	Number of individuals
National Drug Treatment Monitoring System	Treatment clients receiving community or residential structured addiction treatment	Reporting opioid use at triage to treatment	151,983
Drug Test on Arrest	Subjects arrested following a “trigger” offence, or at police discretion, and then tested for opioids and cocaine metabolites by saliva sample, in areas where the drug test on arrest programme operates	Testing positive for opiate use (indicative of recent use)	54,937
Drug Intervention Record	Persons assessed for referral to structured drug treatment following identification in a criminal justice setting	Reporting weekly or greater opioid use at assessment	47,707
Offender Assessment System	Offenders assessed in prison, or whilst on probation for needs and risks prior to sentencing, and at various points throughout their sentence, including assessment of substance use	Reporting weekly or greater opioid use at assessment	39,517

