# Supplementary material for: National record linkage study of mortality for a large cohort of opioid users ascertained by drug treatment or criminal justice sources in England, 2005–2009
Source: Drug Alcohol Depend. 2015 Jan 1;146:17–23. doi: 10.1016/j.drugalcdep.2014.09.782 (PMC4294586; doi:10.1016/j.drugalcdep.2014.09.782)
Supplement: Supplementary file 1 [file mmc1.docx]

**Supplementary material for the article**

**National record linkage study of mortality for a large cohort of opioid users ascertained by drug treatment or criminal justice sources, 2005-2009.**

Matthias Pierce, Sheila M Bird, Matthew Hickman, Tim Millar

Matthias Pierce

Affiliations(s): Institute of Brain Behaviour & Mental Health, Faculty of Medical and Human Sciences, University of Manchester;

Address: NDEC, University of Manchester, 4th Floor, Ellen Wilkinson Building, Oxford Road, UK, M13 9PL

Sheila M. Bird

Affiliation(s): Medical Research Council, Cambridge; University of Strathclyde

Address: MRC Biostatistics Unit, Institute of Public Health, University Forvie Site, Robinson Way, Cambridge. UK, CB2 0SR

Matthew Hickman

Affiliation(s): School of Social and Community Medicine, University of Bristol

Address: School of Social and Community Medicine, University of Bristol, Canynge Hall, 39 Whatley Road, Bristol, UK, BS8 2PS

**Corresponding Author:**

Tim Millar

Affiliations(s): Institute of Brain Behaviour & Mental Health, Faculty of Medical and Human Sciences, University of Manchester

Full Address: NDEC, University of Manchester, 4th Floor, Ellen Wilkinson Building, Oxford Road, UK, M13 9PL

Email: [tim.millar@manchester.ac.uk](mailto:tim.millar@manchester.ac.uk)

Fax: +44 (0)161 275 1668

Telephone: +44 (0)161 275 1661

**This material supplements but does not replace the content of the peer-reviewed paper published in “Drug and Alcohol Dependence”.**

**Analysis of the treatment-subcohort (n= 151,983)**

For the treatment-seeking sub-cohort quality assured variables on potential risk factors were available. Unadjusted and adjusted hazard ratios were calculated, using Cox proportional hazards models, to explore the effect of age-group, gender (and their interaction), and key behavioural risk factors on drug-related poisoning mortality. Subjects entered the risk set on 1st April 2005 or the date of the first treatment triage after which opioid use was declared. Risk period ceased on March 31st 2009 or date of prior death.

Drug use and injecting risk status were acquired from the first triage during the observation period (or 1st April 2005 for prior triages) at which they were declared and the risk was taken to remain until the end of observation. If not recorded, injecting status remained ‘missing’ unless/until updated via a later triage. Only positive responses to other drug misuse variables were recorded, thus missing data could not be quantified for these. Divergence from the proportional hazard assumption was explored using the Schoenfeld residuals.

**Table: CMRs and proportional hazard regression analysis, drug-related poisoning deaths, for the treated opioid user sub-cohort of 151,983 individuals and 442,950 person years**

|  | **Pys**  **(k)** | **Observed deaths** | **CMR** | | **Unadjusted**  **hazard ratio**  **[95% CI]** | **p value** | **Adjusted***  **hazard ratio**  **[95% CI]** | **p value** |
| --- | --- | --- | --- | --- | --- | --- | --- | --- |
| Age |  |  |  |  | |  |  |  |
| 18-34 | 236 | 608 | 26 | 1 | | <0.001 | - |  |
| 35-44 | 150 | 608 | 41 | 1.57 [1.41, 1.76] | |  | - |  |
| 45-64 | 57 | 283 | 50 | 1.91 [1.65, 2.19] | |  | - |  |
| Gender |  |  |  |  | |  |  |  |
| Male | 303 | 1,162 | 38 | 1 | | <0.001 | - |  |
| Female | 140 | 337 | 24 | 0.63 [0.56, 0.71] | |  | - |  |
| Gender and age interaction |  | Test for interaction chi-squared =16.5 (2 dof) p<0.001 | | | | | | |
| Male, 18-34 | 153 | 480 | 31 | - | |  | 1 | <0.001 |
| Male, 35-44 | 108 | 463 | 43 | - | |  | 1.36 [1.20, 1.55] |  |
| Male, 45-64 | 42 | 219 | 52 | - | |  | 1.73 [1.47, 2.03] |  |
| Female, 18-34 | 83 | 128 | 15 | - | |  | 0.51 [0.42, 0.62] |  |
| Female, 35-44 | 42 | 145 | 35 | - | |  | 1.17 [0.97, 1.40] |  |
| Female, 45-64 | 15 | 64 | 43 | - | |  | 1.48 [1.14, 1.93] |  |
| Injecting, ever declared** |  |  |  |  | |  |  |  |
| Yes | 163 | 788 | 48 | 2.15 [1.93, 2.40] | | <0.001 | 2.05 [1.83, 2.29] | <0.001 |
| No | 235 | 526 | 22 | 1 | |  | 1 |  |
| Undeclared | 45 | 185 | 41 | 1.81 [1.53, 2.14] | |  | 1.90 [1.60, 2.25] |  |
| Declared alcohol misuse** |  |  |  |  | |  |  |  |
| Yes | 55 | 313 | 56 | 1.85 [1.64, 2.10] | | <0.001 | 1.70 [1.50, 1.93] | <0.001 |
| No | 387 | 1,186 | 31 | 1 | |  | 1 |  |
| Declared benzodiazepine misuse** |  |  |  |  | |  |  |  |
| Yes | 60 | 288 | 48 | 1.53 [1.35, 1.74] | | <0.001 | 1.38 [1.21, 1.58] | <0.001 |
| No | 383 | 1,211 | 32 | 1 | |  | 1 |  |
| Declared crack cocaine misuse** |  |  |  |  | |  |  |  |
| Yes | 162 | 559 | 34 | 1.03 [0.93, 1.15] | | 0.51 | 0.99 [0.88, 1.10] | 0.79 |
| No | 280 | 940 | 34 | 1 | |  | 1 |  |
| Declared cocaine/ amphetamine misuse** |  |  |  |  | |  |  |  |
| Yes | 58 | 227 | 39 | 1.19 [1.03, 1.37] | | 0.02 | 1.09 [0.95, 1.26] | 0.23 |
| No | 385 | 1,272 | 33 | 1 | |  | 1 |  |

1 indicates baseline category; CMR = crude mortality rate, per 10,000 person years

* Controlling for all other variables in the table

**Entering risk set from date first declared at triage, and subjects remain in that risk set until end of follow-up
